# Unique Gut Microbiome Signatures Depict Diet-Versus Genetically Induced Obesity in Mice

**DOI:** 10.3390/ijms21103434

**Published:** 2020-05-13

**Authors:** Ravinder Nagpal, Sidharth P Mishra, Hariom Yadav

**Affiliations:** 1 Department of Internal Medicine-Molecular Medicine, Wake Forest School of Medicine, Winston-Salem, NC 27101, USA; rnagpal@wakehealth.edu (R.N.); spmishra@wakehealth.edu (S.P.M.); 2 Department of Microbiology and Immunology, Wake Forest School of Medicine, Winston-Salem, NC 27101, USA

**Keywords:** microbiota, obesity, Type 2 diabetes, leptin, diet

## Abstract

The gut microbiome plays an important role in obesity and Type 2 diabetes (T2D); however, it remains unclear whether the gut microbiome could clarify the dietary versus genetic origin of these ailments. Moreover, studies examining the gut microbiome in diet- versus genetically induced obesity/T2D in the same experimental set-up are lacking. We herein characterized the gut microbiomes in three of the most widely used mouse models of obesity/T2D, i.e., genetically induced (leptin-deficient i.e., Lep^ob/ob^; and leptin-receptor-deficient i.e., Lep^db/db^) and high-fat diet (HFD)-induced obese (DIO)/T2D mice, with reference to their normal chow-fed (NC) and low-fat-diet-fed (LF) control counterparts. In terms of β-diversity, Lep^ob/ob^ and Lep^db/db^ mice showed similarity to NC mice, whereas DIO and LF mice appeared as distinct clusters. The phylum- and genus-level compositions were relatively similar in NC, Lep^ob/ob^, and Lep^db/db^ mice, whereas DIO and LF mice demonstrated distinct compositions. Further analyses revealed several unique bacterial taxa, metagenomic functional features, and their correlation patterns in these models. The data revealed that obesity/T2D driven by diet as opposed to genetics presents distinct gut microbiome signatures enriched with distinct functional capacities, and indicated that these signatures can distinguish diet- versus genetically induced obesity/T2D and, if extrapolated to humans, might offer translational potential in devising dietary and/or genetics-based therapies against these maladies.

## 1. Introduction

The prevalence of metabolic diseases, including obesity and Type 2 diabetes (T2D), is rapidly increasing worldwide. Since obesity and T2D co-occur and involve multiple physiological functions associated with the intestinal physiology and ecosystem, specific perturbations in the gut microbial ecosystem (gut dysbiosis) are commonly observed in these diseases. Emerging evidence has highlighted the role of multiple factors including host genetics, nutrition, lifestyle, and microbiome in the pathology of obesity/T2D, with a growing body of evidence underpinning the causal and mechanistic role of the gut microbiome in the development and persistence of these diseases [1,2,3,4]. However, it remains unclear whether the composition and function of the gut microbiome differ in the pathology of diet- vs. genetically induced obesity/T2D. Among various animal models that have been developed and are routinely used to study the pathogenesis and mechanisms of obesity/T2D, high-fat-diet-induced obese (DIO) mice and genetically induced obese mice, i.e., leptin-deficient (Lep^ob/ob^; hereafter, ObOb) mice and leptin-receptor-deficient (Lep^db/db^; hereafter, DbDb) mice remain the most widely used experimental models. However, it remains unexplored whether and how the magnitude and array of gut dysbiosis varies within these different models of obesity/T2D. Specifically, the relative association of the gut microbiome with diet- versus genetically induced obesity/T2D remains unclear. While several previous studies have separately examined the gut microbiome in these models of obesity/T2D [5,6,7,8,9,10], the comparison of these models with each other as well as with their normal chow (NC)-fed and low-fat diet (LFD)-fed counterparts in the same experimental set-up remain unstudied. In particular, considering the emerging speculation that an “obese/T2D microbiome” could play a contributing role in obesity/T2D-associated metabolic dysfunctions, the understanding of gut microbiome composition in different mouse models of obesity/T2D is of considerable research interest and importance. In addition, the differentiation of microbiome signatures specific to host diet and genetics remains highly important in translational applications. We herein examined the gut microbiome compositions in DIO, ObOb, and DbDb mice while also comparing these with NC and LFD controls. The composition of the gut microbiome can be influenced by animal facilities, protocols for fecal collection, DNA isolation and sequencing, and bioinformatics analyses; therefore, comparing the relationship and contribution of gut microbiome composition as determined using data from these mouse models analyzed by different labs/facilities and using different sequencing platforms remains a limitation. To our knowledge, this is the first report to compare the gut microbiome signature in these widely used mouse models of obesity/T2D using identical and consistent protocols and analysis tools, and these results should facilitate prospective studies examining the contribution of the gut microbiome in genetically versus diet-induced pathobiology of obesity/T2D.

## 2. Results

### 2.1. Diet-Induced and Genetically Induced Obese Mice Harbor Distinct Gut Microbiomes

**Figure 1:**
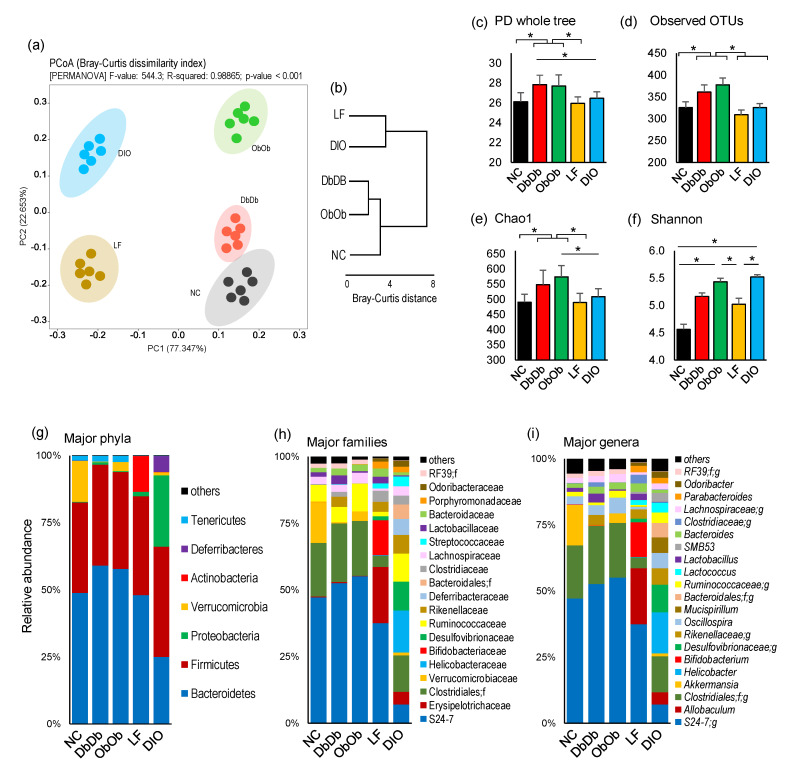
Diet-induced versus genetically induced obese mice exhibit distinct gut microbiome signatures. (**a**) Beta-diversity (principal coordinate analysis; Bray-Curtis dissimilarity index), (**b**) intergroup Bray–Curtis distance, (**c**–**f**) alpha-diversity indices, and (**g**–**i**) microbiome composition at phylum, family, and genus levels in normal-chow-fed leptin-deficient (Lep^ob/ob^, ObOb) and leptin-receptor-deficient (Lepr^db/db^; DbDb) mice and high-fat-diet-fed (diet-induced obese; DIO) mice versus their normal-chow-fed (NC) and low-fat-diet-fed (LF) control counterparts. * *p* < 0.05.

The analysis of β-diversity demonstrated distinct signatures of the fecal microbiome in all five groups of mouse models (Figure 1a). As evident from the principal coordinate analysis (PCoA) analysis of the Bray–Curtis dissimilarity index (Figure 1a,b), the DbDb and ObOb mice clustered closely together and were relatively closer to the NC mice, whereas the LF and DIO mice stood out as separate clusters. The analysis of unweighted and weighted unifrac distance metrics also demonstrated similar patterns of clustering (Supplementary Figure S1). The α-diversity indices showed similar species richness in terms of phylogenetic diversity (PD whole tree), observed operational taxonomic units (OTUs), and Chao1 (species richness) index in NC, LF, and DIO mice, while these indices were significantly or insignificantly higher in DbDb and ObOb mice (*p* < 0.05 versus NC and LF) (Figure 1c–e). On the other hand, the species evenness (Shannon index) is significantly higher (*p* < 0.05) in DIO and ObOb mice as compared to both NC and LF mice (Figure 1f). At the phylum level, the microbiome composition appeared to be relatively similar in NC, DbDb, and ObOb mice, whereas LF and DIO mice demonstrated distinct composition compared to each other as well as compared to the other three groups (Figure 1g). NC mice had a significantly higher (*p* < 0.05) abundance of Verrucomicrobia than all other groups; LF mice had a significantly higher proportion (*p* < 0.05) of Actinobacteria; DIO mice had a significantly higher (*p* < 0.05) proportion of Firmicutes, Proteobacteria, and Deferribacteres; and DbDb and ObOb mice have similar compositions predominated by Bacteroidetes (Figure 1g). Similar compositional patterns were seen at the bacterial family level as well as at the genus level (Figure 1h,i).

**Figure 2:**
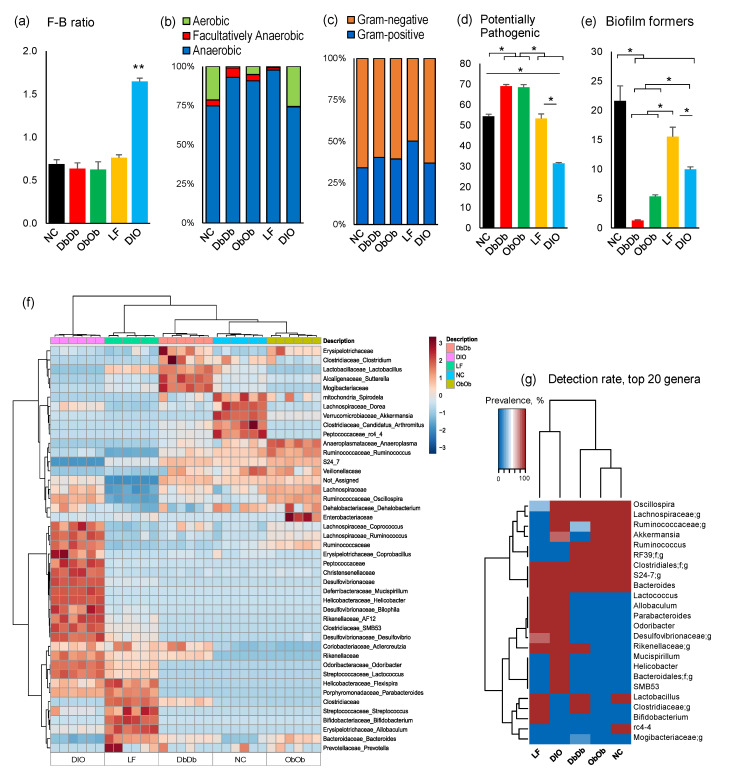
Distinct gut microbiome community structures in diet-induced versus genetically induced obese mice. (**a**) Ratio of major phyla Firmicutes to Bacteroidetes, (**b**) anaerobic to aerobic bacteria, (**c**) Gram-negative to Gram-positive bacteria, (**d**) abundance of bacterial taxa with potential pathogenic phenotypes, (**e**) abundance of bacterial taxa with biofilm-forming traits, (**f**,**g**) heat-map depicting the abundance (**f**) and detection rates (**g**) of major bacterial taxa in normal-chow-fed leptin-deficient (ObOb) and leptin-receptor-deficient (DbDb) mice and high-fat-diet-fed (diet-induced obese; DIO) mice versus their normal-chow-fed (NC) and low-fat-diet-fed (LF) control counterparts. * *p* < 0.05; ** *p* < 0.01.

The overall ratio of the predominant phyla *Firmicutes*: *Bacteroidetes* was remarkably higher (*p* < 0.01) in DIO mice compared to all four other groups (Figure 2a). Analysis of organism-level phenotypes showed that the NC and DIO mice had a significantly higher (*p* < 0.05) ratio of aerobes to anaerobes than the other three groups (Figure 2b). The ratio of Gram-positive to -negative bacteria remained similar in NC, DIO, DbDb, and ObOb mice, but was significantly higher (*p* < 0.05) in LF mice (Figure 2c). Interestingly, the proportion of potentially pathogenic bacteria was significantly (*p* < 0.05) higher in DbDb and ObOb mice and lower in DIO mice than in NC and LF mice (Figure 2d). The abundance of bacteria capable of forming biofilms was significantly (*p* < 0.05) lower in DIO, DbDb, and ObOb mice than in NC and LF mice (Figure 2e). Hierarchical clustering analysis of major bacterial families and genera also demonstrated clearly distinct clusters segregated according to the different groups of mice (Figure 2f), wherein ObOb and DbDb were clustered closer to the NC mice whereas LF and DIO mice presented distinct clusters both from each other and from NC, ObOb, and DbDb mice. Correspondingly, hierarchical clustering analysis in terms of the rate of detection of major genera among these mice also demonstrated a similar arrangement of the clusters, wherein ObOb and DbDb mice clustered closer to the NC mice while LF and DIO mice clustered distinctly (Figure 2g).

### 2.2. Diet-Induced Versus Genetically Induced Obese Mice Demonstrate Unique Signatures of Gut Microbiome Community Structure and Function

**Figure 3:**
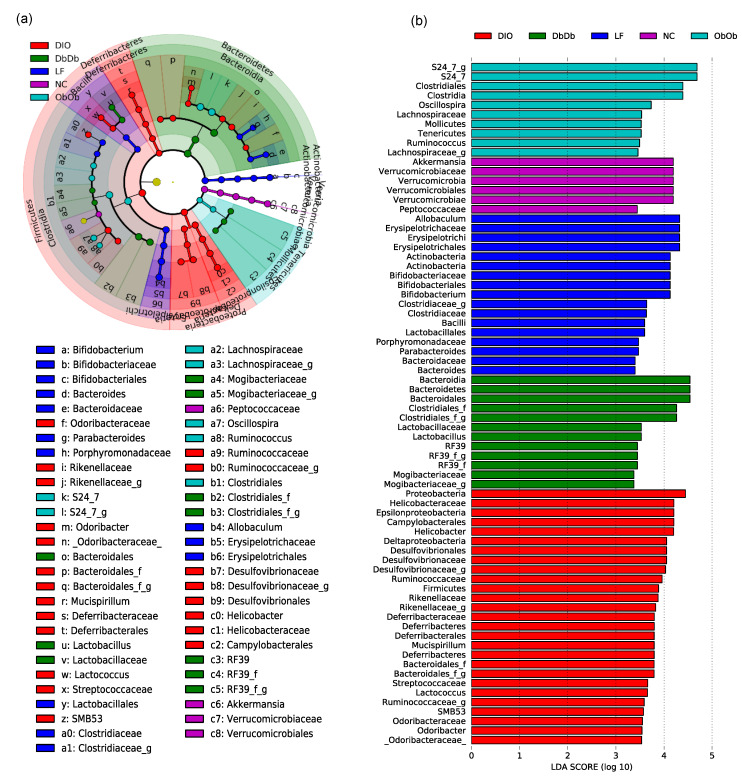
Unique gut microbiome signatures in diet-induced versus genetically induced obese mice. Linear discriminatory analysis (LDA) effect size (LEfSe) analysis cladogram (**a**) and LDA score graph (**b**) illustrating unique bacterial taxa that were differentially and significantly over- or underrepresented (or differentially abundant) and drove differences in normal-chow-fed leptin-deficient (Lep^ob/ob^, ObOb) and leptin-receptor-deficient (Lepr^db/db^; DbDb) mice, high-fat-diet-fed (diet-induced obese; DIO) mice, as well as in normal-chow-fed (NC) and low-fat-diet-fed (LF) control mice.

We then applied linear discriminatory analysis (LDA) effect size (LEfSe) analysis to the inferred relative abundance data to detect unique taxonomic clades and KEGG (Kyoto Encyclopedia of Genes and Genomes) metagenome orthologs that were significantly (*p* < 0.01) over- or underrepresented (or differentially abundant) and could be considered potential metagenomic biomarkers in these different groups of mice. The statistical analysis of the abundance of these bacterial taxa via LEfSe in the form of a cladogram revealed several unique families and genera that drive differences between these five groups of mice (Figure 3a). Figure 3b further simplifies these data and presents the LDA scores of the genera that were significantly (*p* < 0.01) different among these groups (Figure 3b). NC mice were distinguished by higher proportions of phylum Verrucomicrobia, genus *Akkermansia*, and family Peptococcaceae, while LF mice presented a higher proportion of the genera *Allobaculum*, *Bifidobacterium*, *Parabacteroides*, and *Bacteroides*. In contrast, ObOb mice were characterized by a higher abundance of families S24-7 and Lachnospiraceae, order Clostridiales and genera *Oscillospira*, *Tenericutes*, and *Ruminococcus*; whereas DbDb mice were unique in terms of higher carriage of phylum Bacteroidetes, genus *Lactobacillus*, and unclassified genera belonging to the orders Clostridiales and RF39 and the family Mogibacteriaceae. On the other hand, DIO mice harbored the highest proportion of many bacterial taxa belonging to Firmicutes, Proteobacteria, *Helicobacter, Desulfovibrio, Rikenella, Deferribacter, Mucispirillum, Lactococcus, Coprococcus, SMB53*, and *Odoribacter* (Figure 3a,b).

**Figure 4:**
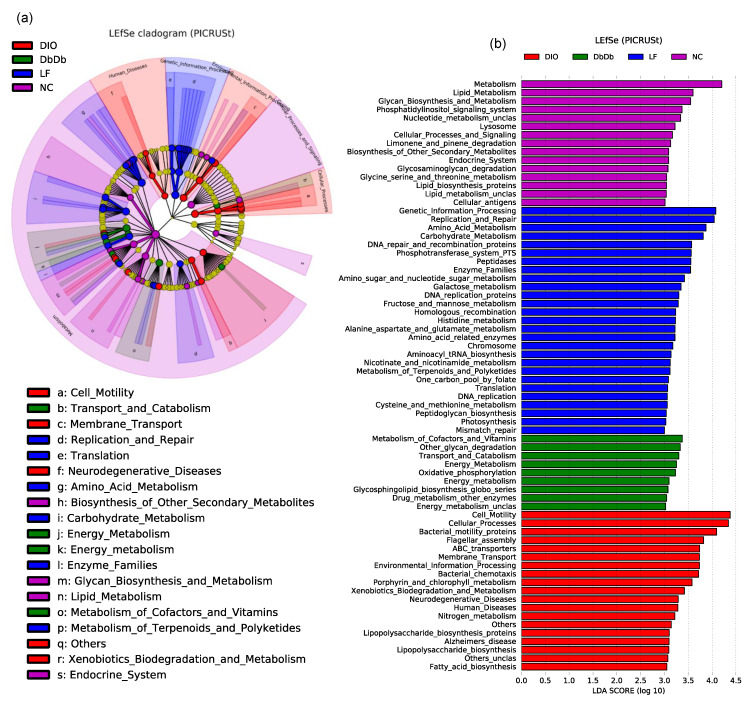
Unique signatures of gut-microbiome-associated metagenomic functions in diet-induced versus genetically induced obese mice. Linear discriminatory analysis (LDA) effect size (LEfSe) analysis cladogram (**a**) and LDA score graph (**b**) illustrating unique metagenomic functional features, as predicted by PICRUSt (Phylogenetic Investigation of Communities by Reconstruction of Unobserved States), that were uniquely and significantly over- or underrepresented (or differentially abundant) and drove differences in normal-chow-fed leptin-deficient (Lep^ob/ob^, ObOb) and leptin-receptor-deficient (Lepr^db/db^; DbDb) mice, high-fat-diet-fed (diet-induced obese; DIO) mice, as well as in normal-chow-fed (NC) and low-fat-diet-fed (LF) control mice.

The PICRUSt (Phylogenetic Investigation of Communities by Reconstruction of Unobserved States) analysis of the KEGG orthologs associated with these bacterial signatures also revealed distinct clusters among these five groups (Figure 4a,b). As evident from the PCoA analysis of the microbiome-associated functional pathways, the DbDb and ObOb mice clustered closely together and were quite similar to the NC mice, whereas the LF and DIO mice stood out as distinct clusters and were highly dissimilar to each other as well as to the other three groups of mice (Supplementary Figure S2a,b). The features were subsequently clustered into functional modules at Levels 1 to 3, as described elsewhere [11]. Figure 4a presents unique features of the Level 1 and 2 modules, while Level 3 features are shown as a bar graph of LDA scores in Figure 4b. The cladogram of LEfSe analysis of the functional Levels 1 and 2 of these annotations clearly reveals distinct arrays of these functions among the different groups (Figure 4a). As further simplified by the LEfSe bar graph, DbDb mice were characterized by a significantly higher (*p* < 0.01) abundance of bacterial taxa associated with the metabolism of cofactors and vitamins, glycan degradation, energy metabolism, and biosynthesis of glycosphingolipids (Figure 4b). In contrast, DIO mice harbored a higher (*p* < 0.01) proportion of bacterial taxa associated with the metabolism of bacterial motility proteins, membrane transporters, metabolism of porphyrin and xenobiotics, biosynthesis of lipopolysaccharides and fatty acids, and the OTUs associated with human diseases including neurodegenerative disease, e.g., Alzheimer’s disease (Figure 4b). In contrast, bacteria associated with glycan and lipid biosynthesis, limonene and glycosaminoglycan degradation, carbohydrate and amino acid metabolism, peptidoglycan biosynthesis, etc. were overrepresented (*p* < 0.01) in NC and LF mice, which means that all of these pathways were less represented (*p* < 0.01) in all three experimental groups of mice. The analysis of the metabolism-related functions alone is presented separately in Supplementary Figures S3 and S4.

**Figure 5:**
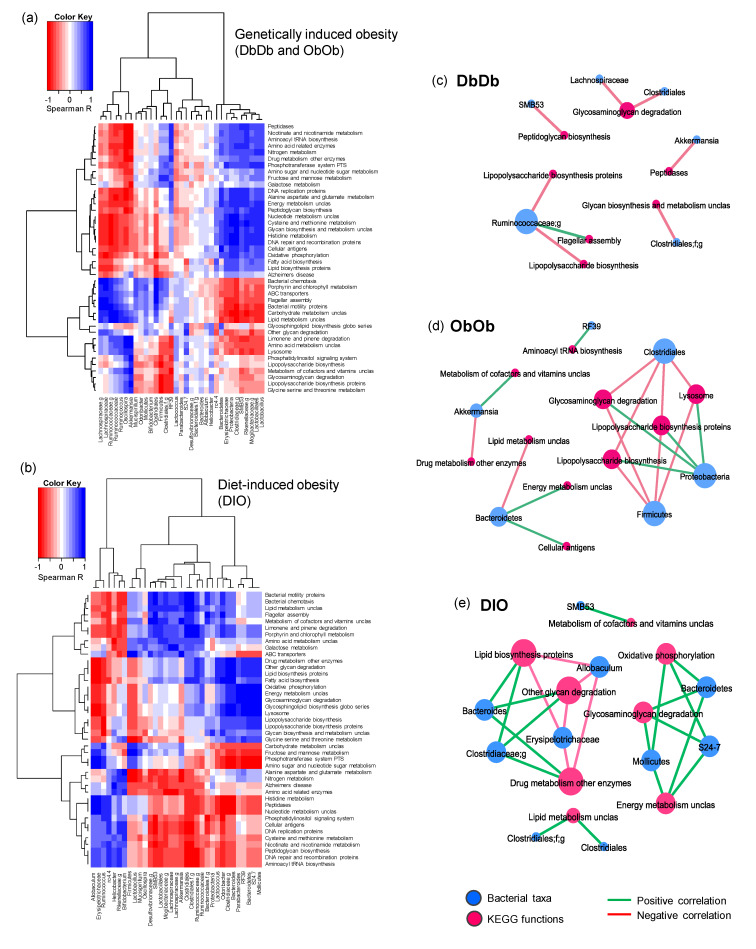
Distinct patterns of correlation between gut bacterial taxa and predicted metagenomic functional features in diet-induced versus genetically induced obese mice. (**a**,**b**) Color heat map depicting the Spearman’s correlation of bacterial taxa identified via amplicon sequencing with PICRUSt-curated metagenomic functions in normal-chow-fed leptin-deficient (Lep^ob/ob^, ObOb) and leptin-receptor-deficient (Lepr^db/db^; DbDb) mice (a) and high-fat-diet-induced obese (DIO) mice (b). Correlation networks showing the selected subsets of significant correlations (Spearman’s correlation rank > 0.7, *p*-value < 0.01) between bacterial taxa and predicted metagenomic functions in (**c**) DbDb, (**d**) ObOb, and (**e**) DIO mice.

We then estimated the relationship between taxonomic and functional enrichments in each obese mouse model by computing the correlations between the abundances of taxonomic clades and KEGG orthologs (metagenomic gene families) using the non-parametric test of Spearman’s rank correlation. The correlations between bacterial abundance and functions enriched in different mouse models were computed via a statistical approach similar to that described by Segata et al. [12]. However, because of the two independent control groups, i.e., NC as a control group for DbDb and ObOb mice (all chow-fed) and LF mice as control group for DIO mice (low-fat- versus high-fat-fed), we first normalized the DbDb and ObOb datasets against NC dataset, while the DIO dataset was normalized against the LF dataset in terms of the fold-difference in the experimental versus control group. The correlation results as depicted in the form of hierarchal heat map yielded clearly distinct clusters of positive and negative associations between a number of bacterial taxa and metagenomic functions in both diet- and genetically induced obese models (Figure 5a–b). To further simplify these clusters, we extracted the significant correlation subsets (Spearman’s rho > 0.7 and *p*-value < 0.01) and built separate correlation networks for each obese model (Figure 5c–e), which showed several common as well as distinct co-occurrence network arrays among these three obese models. The correlation arrays and networks of the complete un-normalized dataset with all five groups combined are presented in Supplementary Figures S5 and S6.

### 2.3. Diet-Induced and Genetically Induced Obese Mice Exhibit Higher Intestinal Permeability and Inflammation

**Figure 6:**
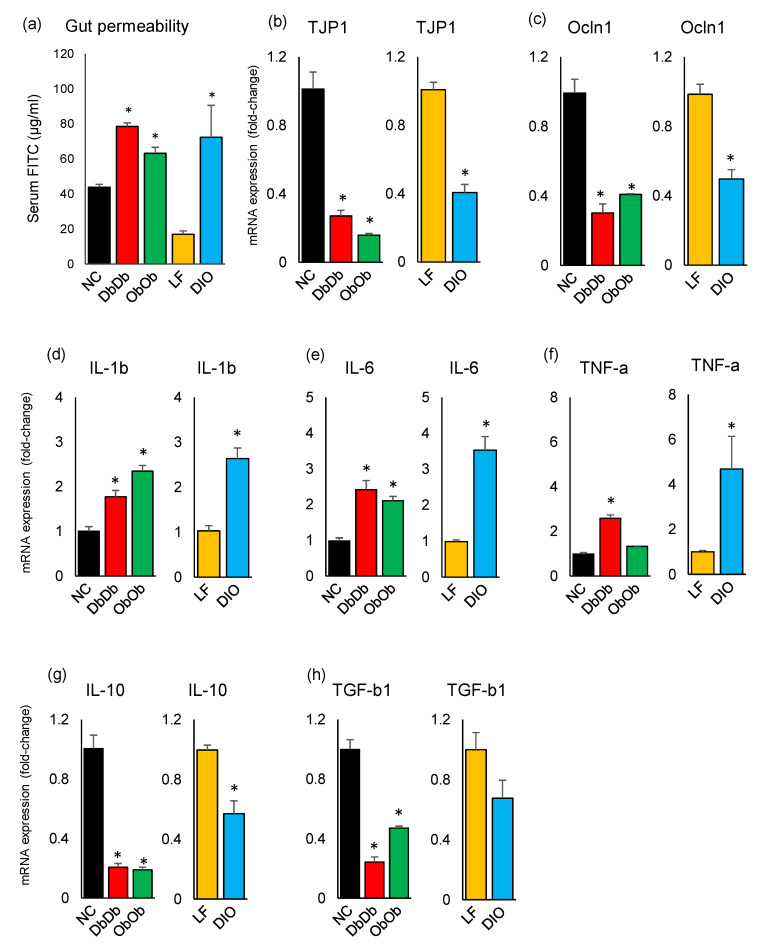
Dysbiotic magnitude of intestinal permeability and inflammatory markers in diet-induced versus genetically induced obese mice. (**a**) Leaky gut (measured by appearance of fluorescein isothiocyanate in blood leaked from gut) (a) and the mRNA expression levels of tight-junction proteins (**b**,**c**) and inflammatory markers (**d**–**h**) in the ileal tissues of chow-fed leptin-deficient (ObOb) and leptin-receptor-deficient (DbDb) obese mice and high-fat-diet-induced obese (DIO) mice, with reference to normal-chow-fed (NC) and low-fat-diet-fed (LF) control mice. * *p* < 0.05.

The gut permeability, as measured by the assayed serum level of fluorescein isothiocyanate (FITC)-dextran (µg/mL serum) 4 h after oral gavage, revealed significantly higher (*p* < 0.05) gut permeability in all three experimental obese models, i.e., DbDb, ObOb, and DIO mice, as compared to their NC and LF counterparts (Figure 6a). Interestingly, LF mice demonstrated the lowest degree of gut permeability, which was insignificantly but considerably lower than even the NC mice. In line with the gut permeability data, the mRNA expression level of epithelial intercellular-tight-junction-associated proteins, i.e., TJP-1 (tight-junction protein-1; also known as Zonula occludens-1 [ZO-1]) and Occl-1 (Occludin-1) was also found to be significantly lower (*p* < 0.05) in all three obese models versus their respective controls (Figure 6b–c). In addition, compared to the respective controls, all three obese models demonstrated significantly higher (*p* < 0.05) expression of inflammatory markers interleukin (IL)-1b, IL-6, and tumor necrosis factor (TNF)-α while exhibiting significantly (*p* < 0.05) lower expression of IL-10 and significantly (*p* < 0.05; DbDb and ObOb) or insignificantly (*p* < 0.1; DIO) lower expression of transforming growth factor (TGF)-β1 (Figure 6d–h).

## 3. Discussion

Both diet and genetics play an important role in the pathology of obesity and T2D, and all these together are linked with gut microbiome dysbiosis. However, it remains unclear whether the gut microbiome can be used to distinguish the diet-versus genetics-specific pathology of these diseases. We herein used high-fat-diet-induced obese (DIO) mice and the genetically induced (ObOb and DbDb) obese/T2D mouse models to determine whether the gut microbiome can disentangle diet- versus genetically induced pathology. To our knowledge, this is the first study demonstrating the gut microbiome composition in the three most widely used obese/T2D mouse models in a nearly identical experimental set-up while also comparing these with their normal-chow-fed (NC) and low-fat-diet-fed (LF) control counterparts.

As revealed by the β-diversity analysis as well as the hierarchical clustering of major bacterial taxa, the mice within an individual group clustered in very close proximity, demonstrating considerable intragroup conservation of taxonomic composition within the respective groups. However, in terms of intergroup variation, all five groups clustered distinctly, indicating different bacterial community structures when compared with each other. The same pattern was seen in the PCoA analysis of PICRUSt-curated functional data. This indicated that differential availability as well as intake of dietary components might result in favored dependence on certain substrates acquired from different diets, which might have contributed to the characteristically different nature of the microbiomes in these mice. The highest similarity was seen between DbDb, ObOb, and NC mice, whereas DIO and LF mice (plausibly due to their distinct diets) showed the least similarity to each other as well as to DbDb, ObOb, and NC mice (Figure 1a,b; Supplementary Figure S2a). This suggests that the LF and DIO microbiomes were less homogenous for low-fat and high-fat diets, respectively, while indicating relatively inherent similarities in the bacterial community structures of NC, DbDb, and ObOb mice, all of which were maintained on normal chow. Since NC, DbDb, and ObOb groups were all maintained on normal chow while LF and DIO mice were maintained on very different diets, i.e., low-fat diet and high-fat diet, respectively, their clustering patterns corroborated previous reports suggesting that diet may dominate the host genotypic background in shaping the murine gut microbiome [13,14]. However, we found the highest α-diversity (species richness) in DbDb and ObOb mice, as compared to all other groups (Figure 1c–e). A higher α-diversity is generally considered a marker of a healthy gut profile, whereas our data indicated that this may not always be the case, particular in the case of leptin- or leptin-receptor-deficient mice, which are characterized by over-eating of an otherwise normal diet that may eventually lead to a dysbiotic gut microflora with an abnormal overgrowth and overrepresentation of specific bacterial groups that otherwise remain subdominant under normal homeostatic milieus (gut eubiosis). Intriguingly, the species evenness was highest in DIO mice (Figure 1f), which could be explained by the fact that the proportion of certain dominant bacterial taxa, such as those belonging to the phylum *Bacteroidetes*, was lower in these mice, meaning that the overall microbiome composition has a greater number of bacterial taxa than in the other four groups of mice. Likewise, the lower proportion of *Rikenellaceae* and *Lactobacillus* may have accounted for the higher representation of *S24-7, Clostridia, Oscillospira, Terericutes*, and *Rumonococcus*, and hence a higher Shannon index, in ObOb versus NC and LF mice. As expected, the weight gain over the course of 8 week experimental period was highest (*p* < 0.01) in DbDb and ObOb mice followed by DIO mice (*p* < 0.05) as compared to NC and LF mice (Figure S7).

The highest proportion of *Bacteroidetes* being found in DbDb and ObOb mice (Figure 1g) was consistent with separate previous studies reporting higher *Bacteroidetes* abundance in ObOb, DbDb, and NC mice of C57Bl/6J background [6,10,15]. However, we found a remarkably higher abundance of *Firmicutes* and *Proteobacteria* and a lower abundance of *Bacteroidetes* in DIO mice compared to all other groups. A higher *Firmicutes* to *Bacteroidetes* ratio has also previously been associated with obesity and poor glucose tolerance [6,16]. Overall, the control NC and LF mice had similar *Firmicutes* to *Bacteroidetes* proportions and were distinguished mainly in terms of higher abundances of Verrucomicrobia and *Actinobacteria*, respectively. DIO, DbDb, and ObOb obese/T2D mice harbored a very different microbiome composition compared to NC and LF mice, underlining an abnormal (dysbiotic) state of gut microbiome in these obese models. However, unsurprisingly, the microbiome composition differed considerably even among these three models, which may have been associated with their diet (high-fat for DIO vs. normal chow for DbDb and ObOb) and/or genetic background (Lep^Ob/Ob^ and Lep^Db/Db^).

Further examination of the taxonomic apportionment via LEfSe analysis revealed a total of 72 differentially abundant bacterial taxa across all five groups of mice (Figure 3a,b). The largest number of unique taxa was seen for the DIO mice, while the lowest number was recorded for NC mice. This low number of unique taxonomic biomarkers in NC mice may be ascribed to the comparatively higher bacterial community structure similarity between NC, DbDb, and ObOb mice than others, thereby leading to a smaller array of unique and significantly differential taxa. The higher number of unique taxa in DIO followed by LF mice indicated that the preferential abundance of bacterial lineages emanating from the particular higher-level bacterial taxa, probably driven by high-fat and low-fat diets, respectively, led to definitive compositional differences in the gut microbiomes of these mice. Overall, NC mice were characterized by two discriminative features (*Akkermansia* and *Peptococcaceae*) whereas DbDb and ObOb mice presented five and six, respectively, taxonomic features. In contrast, DIO mice presented 11 discriminative features that were uniquely abundant versus all other groups. This clearly suggests that both models (diet- versus genetically induced) of obesity present some degree of microbiome dysbiosis; however, particularly in terms of taxa with altered proportions, a high-fat diet is more perturbing to the microbiome spectrum than genetically induced obesity. In addition, the data from ObOb and DbDb mice might suggest some sort of direct connection between gut epithelial leptin signaling and specific gut microbes. Particularly considering the role of leptin in regulating various processes of metabolism and immunity, it is plausible that leptin activity might influence gut bacterial populations and vice versa, maybe even independently of host dietary intake. Interestingly, in contrast to previous studies reporting a lower proportion of family *Ruminococcaceae* in DIO mice [17], we found a higher abundance of this family in DIO mice This difference may be due to the differences in geographical location of the experimental facilities. In addition, these inconsistencies between different studies reporting the effect of obesity on gut microbiomes suggest the possibility of high model and environmental specificity. Interestingly, compared to NC mice, the abundance of genus *Akkermansia* was significantly lower in DIO, DbDb, and ObOb mice. Notably, *Akkermansia* has been found to play an important role in glucose homeostasis and is being explored as a therapeutically beneficially bacterium to ameliorate insulin resistance and related metabolic disorders [4,18,19,20,21]. The PICRUSt-based analysis of differentially abundant functional metagenome features (classified as KEGG orthologs (KOs)) identified 70 functional KOs across all samples. However, no differentially abundant feature was detected in ObOb mice, which may be a reflection of the comparatively higher similarity between NC and ObOb mice in terms of microbial taxa as well as function modules, thus leading to less magnitude of unique and significantly differential features.

We then computed the correlations between bacterial abundance and functions enriched in different mouse models. The abundance of taxa involved in glycosaminoglycan degradation correlated negatively with that of *Clostridiales* in both DbDb and ObOb mice, but associated positively with *Proteobacteria* in ObOb and with *Bacteroidetes* in DIO mice (Figure 5c–e). Interestingly, the abundance of taxa associated with lipopolysaccharide (LPS) biosynthesis correlated negatively with *Firmicutes* and its members, including *Clostridia* and *Ruminococci*, in both DbDb and ObOb mice. This may be because LPS is a major cell-wall component of Gram-negative bacteria, whereas the majority of *Firmicutes*, which were lower in DbDb and ObOb versus DIO mice, are Gram-positive. The abundance of phylum *Bacteroidetes*, which comprises many clades involved in carbohydrate metabolism, correlated positively with the abundance of taxa involved in energy metabolism in both ObOb and DIO mice. In DIO mice, which were fed a high-fat diet and had a lower abundance of *Bacteroidetes*, the abundance of *Bacteroides* correlated positively with taxa involved in lipid biosynthesis, whereas in ObOb mice, taxa involved in lipid metabolism correlated negatively with *Bacteroidetes*. Interestingly, the higher abundance of taxa involved in LPS biosysnthesis in DIO mice corroborated the data of higher gut permeability and inflammation in these mice, concurring with multiple previous studies reporting the implication of gut leakiness and consequent gut-to-blood translocation of LPS in low-grade inflammation leading to insulin resistance, T2D, and metabolic syndrome in obese mice as well as in human subjects [22,23,24]. Altogether, these correlation network arrays indicated that for these obese mouse models, genomic composition of the bacteriome might be representative of the direct intestinal environment, especially in terms of dietary phenotype, nutrient pattern, and intake, and the energy, vitamin, and lipid metabolism pattern, in addition to other functional features. Our data revealed several pathways that were differentially important in these models and might be used for differentiation between diet-induced versus genetically induced obesity. Further empirical and more mechanistic and inclusive studies would support these markers and pin down functional biomarkers exclusive to obesity-specific biomes. The data also suggested that these taxonomic biomarkers contribute significantly to important gut metagenomic functions and may be determined by their metabolic capabilities. Hence, more inclusive and comprehensive studies that integrate metabolomics data with metagenomic arrays would be of great interest and importance in further validation and elucidating mechanistic implications of specific gut microbiome signatures in diet- versus genetically induced obesity.

Gut hyperpermeability is one of the common causes of high-fat-diet-induced obesity, leading to abnormally increased diffusion and gut-to-blood leakage of various bacterial substances such as LPS through the mucous layer and tight junctions, thereby inciting low-grade inflammation, which is one of the major characteristics of obesity/T2D [22,23,24]. Interestingly, our data revealed higher proportions of bacteria involved in LPS biosynthesis in DIO mice, thereby corroborating these reports. However, the data of higher gut permeability in all three obese models, i.e., DbDb, ObOb, and DIO mice, indicated that obesity/T2D may implicate increased gut permeability (“leaky gut”) independent of host diet, and that this obesity-associated gut hyperpermeability might result in hyperinflammation or endotoxemia even without consuming a HFD. The lower mRNA expression levels of TJP-1 and Occl-1 further suggested some sort of impaired intestinal barrier integrity in the three obese groups. In line with the impaired gut barrier permeability and integrity, we also found higher expression of pro-inflammatory markers IL-1β, IL-6, and TNF-α in all three models. Leaky gut and consequent systemic inflammation is often implicated as one of the causes of obesity/T2D and metabolic syndrome; however, our data suggested that a genetic obese/T2D phenotype without any HFD exposure may also cause the state of gut hyperpermeability. However, it remains unexplained whether the state of hyperinflammation is the cause or consequence of gut leakiness in DbDb and ObOb mice. Further studies should investigate the possible mechanisms underlying this association of leptin and leptin-receptor deficiency with gut dysbiosis, hyperpermeability, and hyperinflammation.

In summary, we herein demonstrated that obesity driven by a high-fat diet versus genetic mutation presents a different gut microbiome composition, hinting that the microbiome is sensitive to both host diet and to genetic background and physiology. We identified several biomarkers, both taxonomic and functional, which may provide new insights into the different obesity/T2D phenotypes through the discovery of important gut bacterial taxa and metabolic pathways in different model systems. The data suggest that diet or obese/T2D phenotypes alone are not the sole driving factors of the metagenomic fabric in these models, even if they may the most predominant ones. Although the study did not examine the mechanisms linking microbiome signatures with the pathophysiology of obesity/T2D as such, the data demonstrated how gut microbiomes and permeability may vary in different models of obesity/T2D. These microbiome variations appear to be model-specific, with divergent functional mechanisms depending on host diet and genetic background. The data should facilitate prospective studies focused on understanding the pathophysiology of obesity/T2D with particular reference to the gut microbiome. It is our belief, particularly in the context of obesity/T2D, that such a comparative understanding of the way microbiomes differ between different experimental models would not only broaden our knowledge of these diseases, but would also facilitate the selection of an appropriate model for a specific investigation in future studies. It would also be interesting for further studies to investigate these model-specific microbiome differences particularly at different stages of the disease progression, as well as to establish whether and how these taxa functionally modulate glucose homeostasis, thereby ultimately providing microbiotic recourse for developing novel therapies against obesity/T2D.

## 4. Materials and Methods

### 4.1. Animals

The study included five groups of C57B6/J male mice (*n* = 6 per group; housed three per cage), i.e., (1) normal-chow-fed (NC), (2) low-fat-diet-fed (LFD), (3) high-fat-diet-fed (diet-induced obese; DIO); (4) normal-chow-fed leptin-deficient (Lep^ob/ob^; ObOb), and (5) normal-chow-fed leptin-receptor-deficient (Lepd^b/db^; DbDb) mice. The latter three are common and widely used mouse models of obesity/T2D. The mice were 5–6 weeks old at the time of enrolment into specific groups. The mice in a given group were from two to three sets of pairs that were littermates from a single breeder pair. All the animals were maintained in the same room with controlled atmosphere of 22 ± 2 °C and 55% ± 10% relative humidity with a 12 h/12 h light/dark cycle. Upon enrolment into specific groups, all the animals were maintained on free access to respective normal chow (NC, ObOb, Dbdb), low-fat (LF; 10kcal% fat, D12450J, Research Diets Inc., New Brunswick, NJ, USA), or high-fat (DIO; 60 kcal% fat, D12450J, Research Diets Inc.) diets for a period of eight weeks, with free access to water. All experiments were performed in accordance with the guidelines of the institutional ethical committee of animal use and care (Protocol number: A17-033, approved on May 30, 2017).

### 4.2. Gut Microbiome Analysis

Gut microbiomes were analyzed per our previously described methods [25,26,27,28]. In brief, the Earth Microbiome Project (EMP) benchmarked protocol [29] (http://www.earthmicrobiome.org/protocols-and-standards/) was followed by employing a barcoded high-throughput sequencing approach, as described in Caporaso et al. [30]. Bacterial genomic DNA was extracted using the MoBio PowerFecal DNA kit (Qiagen, CA, USA). The V4 hypervariable region of the 16S rDNA gene was amplified using the universal primer pair 515F (barcoded) and 806R [30]; resulting uniquely barcoded amplicons were purified using Agencourt^®^ AMPure^®^ XP magnetic purification beads (Beckman Coulter, Brea, CA, USA) and quantified using a Qubit-3 fluorimeter (InVitrogen, Carlsbad, CA, USA) and the dsDNA HS assay kit (Life Technologies, Carlsbad, CA, USA); the amplicon library was generated according to the methods of Caporaso et al. [30]. The purified PCR products were pooled in equal molar concentrations and sequenced on one 2 × 251 bp Illumina MiSeq run (Illumina Miseq reagent kit v3) for paired-end sequencing. The sequencing quality control was executed via onboard Miseq Control Software and Miseq Reporter (Illumina Inc., San Diego, CA, USA). The resulting sequences were de-multiplexed, quality-filtered, clustered, and taxonomically assigned (on the basis of 97% similarity level against the GreenGenes database, May 2013 version) with RDP (Ribosomal Database Project) classifiers as described by Wang et al. [31] by using the QIIME (Quantitative Insights into Microbial Ecology) software package (version 1.9.1; Boulder, CO, USA; qiime.org) [32] as per our previously described workflow [25,28]. To avoid the influence of DNA extraction, PCR conditions, and primers on community composition recovered by amplicon sequencing, all samples were processed simultaneously and identically in order to minimize biasing of the bacterial community composition. Of the total 11,635,114 reads (average depth 38,783 reads/sample) originally obtained, a total of 1,087,990 reads (average depth 36,266 reads/sample) remained after quality control. To avoid bias of sequencing depth, the data were rarefied to lowest sample depth, i.e., 11,639 sequences/sample prior to downstream analyses. To avoid bias of sequencing errors or low-level contaminations, the OTUs with very small counts (fewer than four) in very few samples (less than 10% prevalence) were filtered out from the subsequent analyses, as described by Chong et al. [33]. The taxon abundance data were subjected to total sum scaling and the taxa with less than 0.5% mean relative abundance were further excluded from the subsequent downstream analyses. Bacterial community compositions of each sample were measured at taxonomic levels of phylum, class, order, family, and genus. Alpha-diversity measures were computed within QIIME. Beta-diversity was analyzed using PCoA of the Bray–Curtis dissimilarity index, as described previously [33]. The metabolic and other functional activities of the gut bacterial communities were analyzed using the open source bioinformatics tool PICRUSt against the functional database of KEGG Orthology, as described previously [11].

### 4.3. Gut Permeability Measurement

Gut permeability was measured as per our previously described methods [34,35]. Briefly, mice were fasted for four hours and were then given an oral gavage of fluorescein isothiocyanate (FITC)-dextran (3–5 kDa; 1 g/kg body weight; Millipore Sigma, Burlington, MA, USA). After four hours of gavage (still on fast), the appearance of FITC fluorescence (excitation at 485 nm and emission at 520 nm) was measured (in duplicate from each mouse) in the serum and the gut permeability was calculated with reference to the FITC standard curve, as described previously [34,35].

### 4.4. Real-Time PCR Assays

Gene expression assays of tight-junction proteins and inflammation-related markers were conducted as per our previously described methods [34,35]. Briefly, total RNA was extracted from ileum tissues using an RNeasy kit (Qiagen, Germantown, MD, USA) and was reverse-transcribed using a High-Capacity cDNA reverse transcription kit (Applied Biosystems, Foster City, CA, USA). The cDNA was used to quantify the expression of TJP-1, Occl-1, IL-1β, IL-6, IL-10, TNF-α, and TGF-β1 using TaqMan Gene Expression Assays for real-time PCR. 18S rRNA was used as an internal control. Relative gene expression was calculated using the ^ΔΔ*C*T^ method and presented as relative fold change. All the assays were performed in triplicate and repeated three times.

### 4.5. Data Analysis

Alpha-diversity indices, bacterial abundance, gut permeability, and the gene expression data were compared between different groups using Kruskal–Wallis test followed by pair-wise Mann–Whitney U comparison. Resulting *p*-values were corrected by Bonferroni method. LEfSE (linear discriminatory analysis (LDA) effect size) was performed to identify bacterial taxa and metagenome functional modules driving differences in the different groups of mice [36], wherein the alpha parameter significance threshold for the Kruskal–Wallis as well as the Wilcoxon test implemented among classes was set to 0.01, the logarithmic LDA score cut-off was set to 3, and the strategy for multi-class analysis was set to “all-against-all”. Bray–Curtis dissimilarity scores inferred from the bacteriome taxonomic data were reduced to a two-dimensional space using principal coordinate analysis (PCoA) to estimate the structural similarity (beta-diversity) of bacteriomes from different groups of mice. Differences in beta-diversity were tested by permutational multivariate analysis of variance (PERMANOVA) using the web-based algorithm tool MicrobiomeAnalyst [37]. Hierarchical clustering and heat maps depicting the patterns of abundance and log values were constructed within the “R” statistical software package (version 3.6.0; https://www.r-project.org/) using the “heatmap.2” and “ggplots” packages. Spearman’s correlations of bacterial taxa with KEGG metagenomic functions were calculated in GraphPad Prism software (San Diego, CA, USA; version 6.0; https://www.graphpad.com/scientific-software/prism/). Co-occurrence networks between taxa and functions were calculated by using the open-source software Gephi (https://gephi.org/) to find differential associations caused by similar alterations in the proportion of different taxa and their predicted functions between different groups of mice. Modularity-based co-occurrence networks were analyzed at a Spearman’s correlation cutoff of 0.7 and *p*-value < 0.01; the selected correlation data were imported into the interactive platform, Gephi (version 0.9.2; https://gephi.org), and the following modularity analyses and keystone node identification were conducted within Gephi. Unless otherwise stated, all bar graphs presented herein represent means ± SEM. *p* < 0.05 was considered statistically significant unless otherwise specified.

## Supplementary Materials

Supplementary materials can be found at https://www.mdpi.com/1422-0067/21/10/3434/s1. Supplementary figures have been provided along with this manuscript. All the raw sequencing data sets have been submitted to the NCBI Sequence Read Archive database under SRA accession number: SUB7192037 and bio-project number PRJNA615360).

## Abbreviations

DIO	Diet-induced obesity
ObOb	Leptin-deficient (Lep^ob/ob^)
DbDb	Leptin-receptor-deficient (Lepr^db/db^)
LF	Low-fat
NC	Normal chow
HFD	High-fat diet
T2D	Type-2 diabetes
LDA	Linear discriminatory analysis
LEfSe	Linear discriminatory analysis effect size
KEGG	Kyoto Encyclopedia of Genes and Genomes
KOs	KEGG orthologs
QIIME	Quantitative Insights into Microbial Ecology
PICRUSt	Phylogenetic Investigation of Communities by Reconstruction of Unobserved States
FITC	Fluorescein isothiocyanate
TJP	Tight-junction proteins
IL	Interleukins
TGF	Transforming growth factor
TNF	Tumor necrosis factor
PCoA	Principal coordinate analysis
EMP	Earth Microbiome Project
