# Supplementary material for: Unique Gut Microbiome Signatures Depict Diet-Versus Genetically Induced Obesity in Mice
Source: Int J Mol Sci. 2020 May 13;21(10):3434. doi: 10.3390/ijms21103434 (PMC7279357; doi:10.3390/ijms21103434)
Supplement: Supplementary file 1 [file ijms-21-03434-s001.pdf]

Supplemental data

Unique gut microbiome signatures shaped by diet- versus genetically-induced obesity in mice

Ravinder Nagpal[1], Sidharth Mishra[1], and Hariom Yadav[1,2*]

[1]Department of Internal Medicine-Molecular Medicine, [2]Department of Microbiology and Immunology, Wake Forest School of Medicine, Winston-Salem, NC, USA.

*<u>*Corresponding Author*</u>: Hariom Yadav, PhD, Assistant Professor, Department of Internal Medicine-Molecular Medicine, Department of Microbiology and Immunology, Center for Diabetes, Obesity and Metabolism, Wake Forest School of Medicine, Wake Forest Biotech Place, Suite 2E-034, 575 Patterson Ave., Winston-Salem, NC 27101, USA; Tel: +1-336-713 5049; Email: hyadav@wakehealth.edu

Suppl. Fig. 1

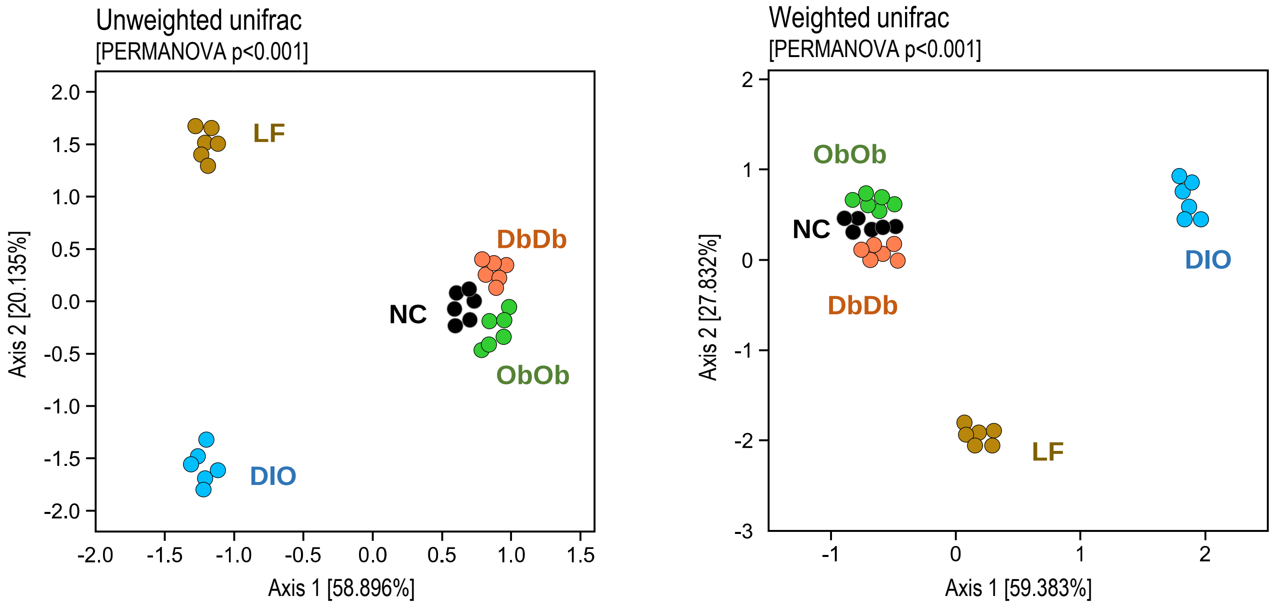

Unweighted unifrac
[PERMANOVA p<0.001]
LF
DbDb
NC
ObOb
DIO
Axis 2 [20.135%]
Axis 1 [58.896%]
2.0
1.5
1.0
0.5
0.0
-0.5
-1.0
-1.5
-2.0
-2.0
-1.5
-1.0
-0.5
0.0
0.5
1.0
1.5
Weighted unifrac
[PERMANOVA p<0.001]
ObOb
NC
DbDb
DIO
LF
Axis 2 [27.832%]
Axis 1 [59.383%]
2
1
0
-1
-2
-3
-1
0
1
2

Suppl. Fig. 2

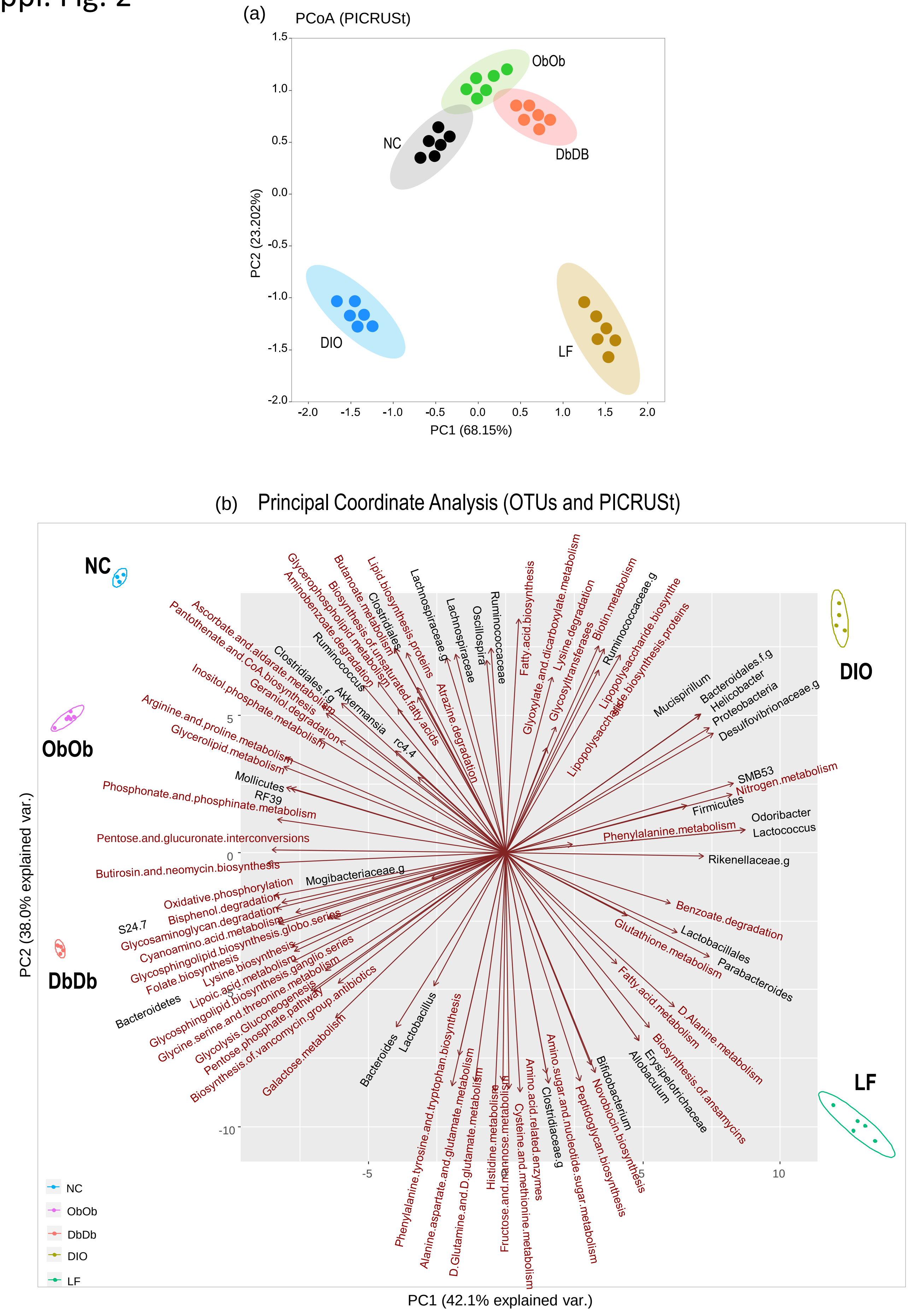
(a)
PCoA (PICRUSt)
ObOb
NC
DbDB
DIO
LF
PC2 (23.202%)
PC1 (68.15%)
1.5
1.0
0.5
0.0
-0.5
-1.0
-1.5
-2.0
-2.0
-1.5
-1.0
-0.5
0.0
0.5
1.0
1.5
2.0
(b)
Principal Coordinate Analysis (OTUs and PICRUSt)
NC
ObOb
DbDb
DIO
LF
PC2 (38.0% explained var.)
PC1 (42.1% explained var.)
Glycerophospholipid.metabolism
Aminobenzoate.degradation
Butanoate.metabolism
Biosynthesis.of.unsaturated.fatty.acids
Lipid.biosynthesis.proteins
Clostridiales
Lachnospiraceae.g
Lachnospiraceae
Oscillospira
Ruminococcaceae
Fatty.acid.biosynthesis
Glyoxylate.and.dicarboxylate.metabolism
Lysine.degradation
Glycosyltransferases
Biotin.metabolism
Ruminococcaceae.g
Lipopolysaccharide.biosynthesis.proteins
Lipopolysaccharide.biosynthesis
Ascorbate.and.aldarate.metabolism
Pantothenate.and.CoA.biosynthesis
Clostridiales.f.g
Ruminococcus
Geraniol.degradation
Inositol.phosphate.metabolism
Akkermansia
rc4.4
Atrazine.degradation
Arginine.and.proline.metabolism
Glycerolipid.metabolism
Mollicutes
RF39
Phosphonate.and.phosphinate.metabolism
Pentose.and.glucuronate.interconversions
Butirosin.and.neomycin.biosynthesis
Mogibacteriaceae.g
Oxidative.phosphorylation
Bisphenol.degradation
S24.7
Glycosaminoglycan.degradation
Cyanoamino.acid.metabolism
Glycosphingolipid.biosynthesis.globo.series
Folate.biosynthesis
Lysine.biosynthesis
Lipoic.acid.metabolism
Glycosphingolipid.biosynthesis.ganglio.series
Glycine.serine.and.threonine.metabolism
Glycolysis.Gluconeogenesis
Pentose.phosphate.pathway
Biosynthesis.of.vancomycin.group.antibiotics
Galactose.metabolism
Bacteroidetes
Bacteroides
Lactobacillus
Phenylalanine.tyrosine.and.tryptophan.biosynthesis
Alanine.aspartate.and.glutamate.metabolism
D.Glutamine.and.D.glutamate.metabolism
Histidine.metabolism
Fructose.and.mannose.metabolism
Cysteine.and.methionine.metabolism
Amino.acid.related.enzymes
Clostridiaceae.g
Amino.sugar.and.nucleotide.sugar.metabolism
Peptidoglycan.biosynthesis
Novobiocin.biosynthesis
Bifidobacterium
Allobaculum
Erysipelotrichaceae
Biosynthesis.of.ansamycins
D.Alanine.metabolism
Fatty.acid.metabolism
Glutathione.metabolism
Parabacteroides
Lactobacillales
Benzoate.degradation
Rikenellaceae.g
Phenylalanine.metabolism
Lactococcus
Odoribacter
Firmicutes
Nitrogen.metabolism
SMB53
Mucispirillum
Bacteroidales.f.g
Helicobacter
Proteobacteria
Desulfovibrionaceae.g
5
0
-5
-10
-5
5
10
NC
ObOb
DbDb
DIO
LF

Suppl. Fig. 3

LEfSe analysis cladogram illustrating unique metagenomic metabolism-related functional features, as predicted by PICRUSt, that are significantly over- or under-represented (or differentially abundant) in ObOb and DbDb mice, DIO mice, as well as in NC and LF control mice.

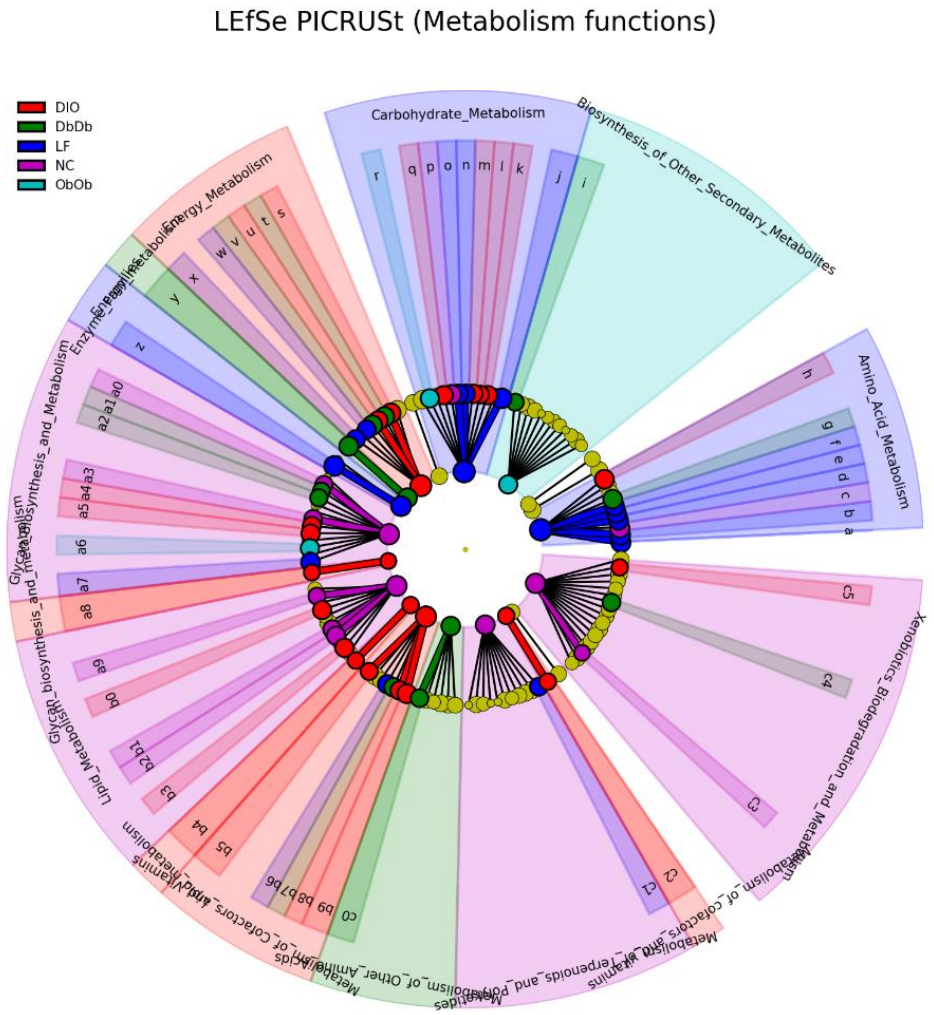

a: Alanine_aspartate_and_glutamate_metabolism
b: Amino_acid_related_enzymes
c: Arginine_and_proline_metabolism
d: Cysteine_and_methionine_metabolism
e: Glycine_serine_and_threonine_metabolism
f: Histidine_metabolism
g: Lysine_biosynthesis
h: Phenylalanine_tyrosine_and_tryptophan_biosynthesis
i: Phenylpropanoid_biosynthesis
j: Amino_sugar_and_nucleotide_sugar_metabolism
k: Butanoate_metabolism
l: C5_Branched_dibasic_acid_metabolism
m: Citrate_cycle_TCA_cycle
n: Fructose_and_mannose_metabolism
o: Galactose_metabolism
p: Glycolysis_Gluconeogenesis
q: Glyoxylate_and_dicarboxylate_metabolism
r: Pentose_and_glucuronate_interconversions
s: Carbon_fixation_pathways_in_prokaryotes
t: Methane_metabolism
u: Nitrogen_metabolism
v: Oxidative_phosphorylation
w: Photosynthesis
x: Photosynthesis_proteins
y: Energy_metabolism_unclas
z: Peptidases

a0: Glycosaminoglycan_degradation
a1: Glycosphingolipid_biosynthesis_ganglio_series
a2: Glycosphingolipid_biosynthesis_globo_series
a3: Glycosyltransferases
a4: Lipopolysaccharide_biosynthesis
a5: Lipopolysaccharide_biosynthesis_proteins
a6: Other_glycan_degradation
a7: Peptidoglycan_biosynthesis
a8: Glycan_biosynthesis_and_metabolism_unclas
a9: Biosynthesis_of_unsaturated_fatty_acids
b0: Fatty_acid_biosynthesis
b1: Glycerolipid_metabolism
b2: Glycerophospholipid_metabolism
b3: Lipid_biosynthesis_proteins
b4: Lipid_metabolism_unclas
b5: Biotin_metabolism
b6: Nicotinate_and_nicotinamide_metabolism
b7: One_carbon_pool_by_folate
b8: Pantothenate_and_CoA_biosynthesis
b9: Porphyrin_and_chlorophyll_metabolism
c0: Cyanoamino_acid_metabolism
c1: Prenyltransferases
c2: Metabolism_of_cofactors_and_vitamins_unclas
c3: Atrazine_degradation
c4: Drug_metabolism_other_enzymes
c5: Nitrotoluene_degradation

Suppl. Fig. 4

LEfSe analysis illustrating the LDA scores of unique metagenomic metabolism-related functional features, as predicted by PICRUSt, that are significantly over- or under-represented (or differentially abundant) in ObOb and DbDb mice, DIO mice, as well as in NC and LF control mice.

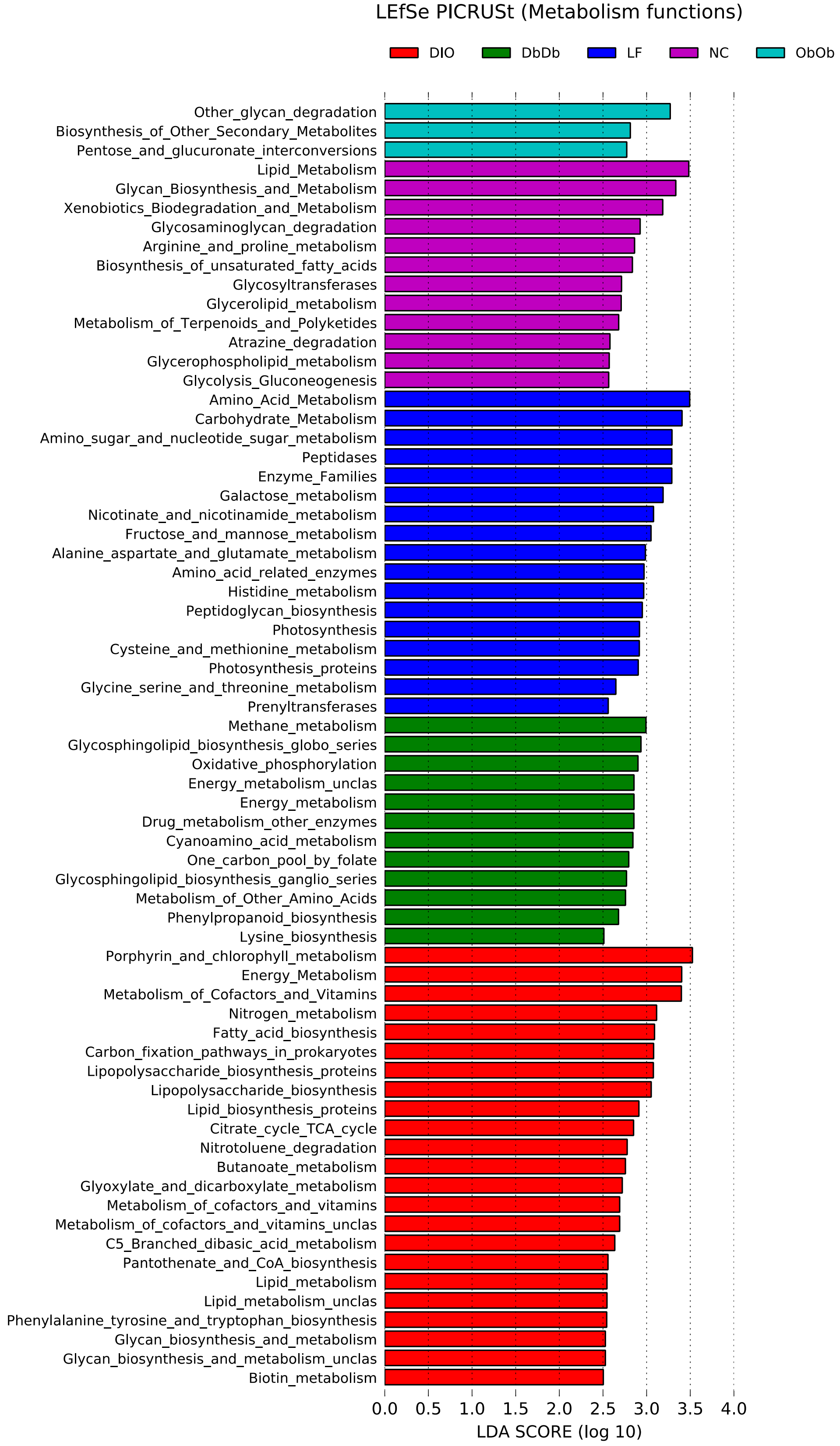

Suppl. Fig. 5

Color heat-map depicting the overall spearman correlation of bacterial taxa with the PICRUSt-curated metagenomic functions altogether in ObOb, DbDb, DIO, NC and LF mice.

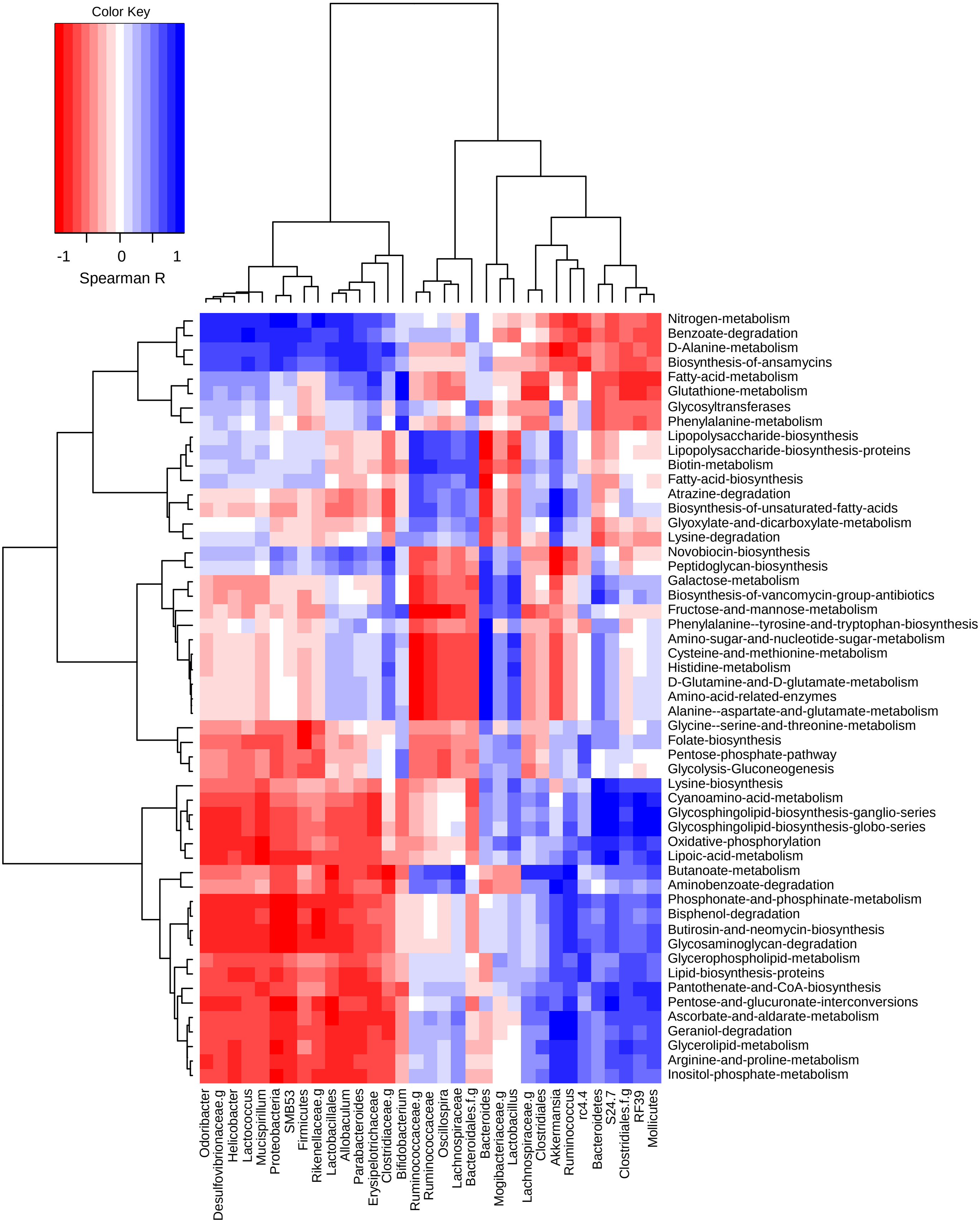

Suppl. Fig. 6

Significant associations with correlation >0.7 and p-value <0.01 (FDR)

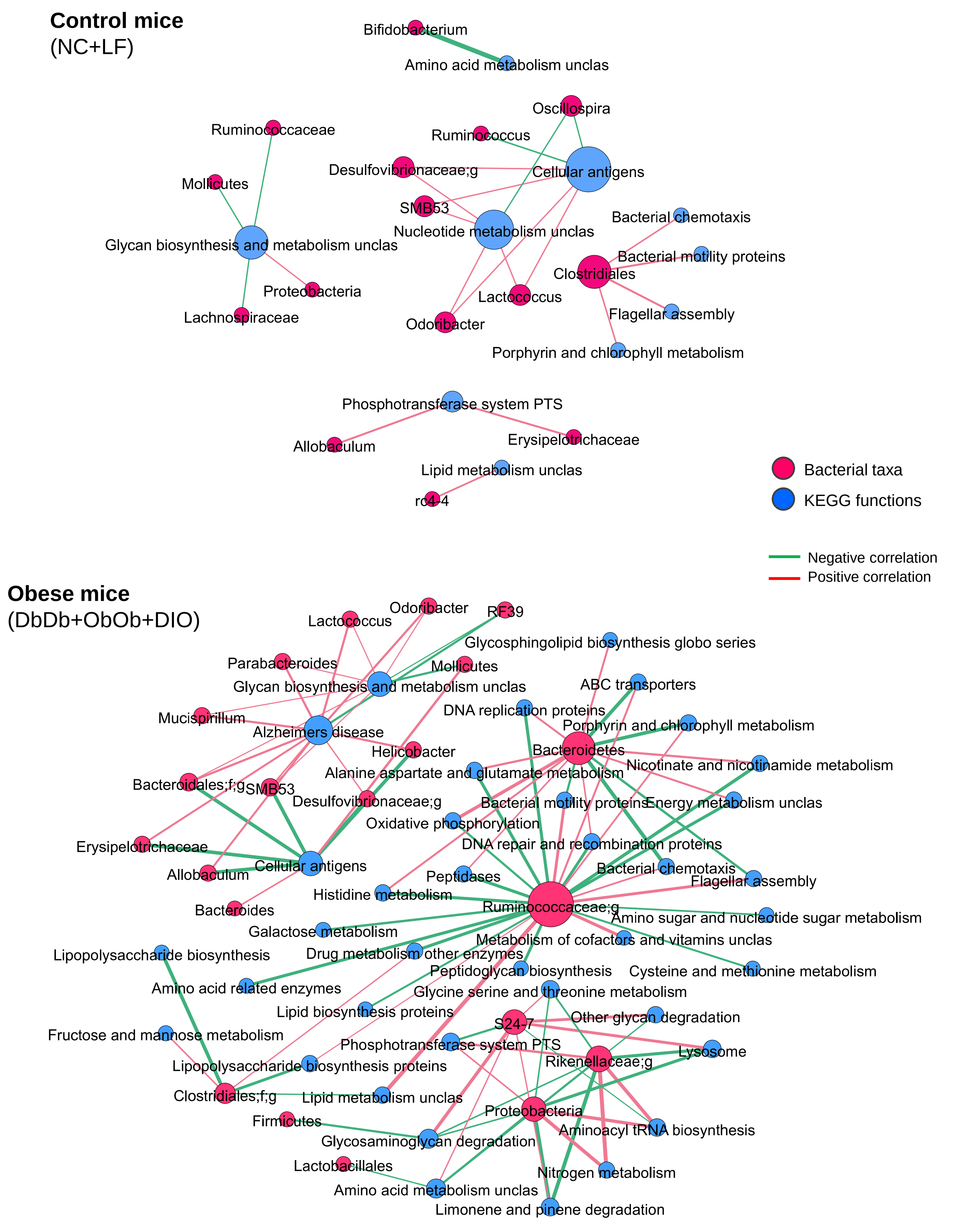

Suppl. Fig. 7

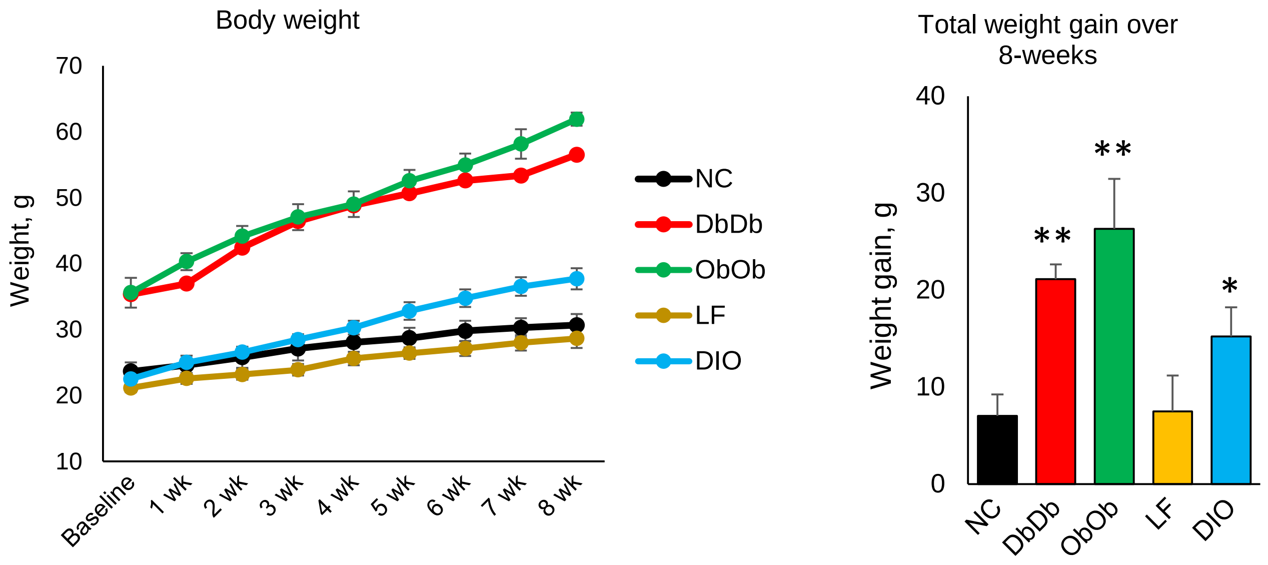
Body weight
70
60
50
40
30
20
10
Weight, g
Baseline
1 wk
2 wk
3 wk
4 wk
5 wk
6 wk
7 wk
8 wk
NC
DbDb
ObOb
LF
DIO
Total weight gain over 8-weeks
40
30
20
10
0
Weight gain, g
**
**
*
NC
DbDb
ObOb
LF
DIO
